# Supplementary material for: Chromoblastomycosis and phaeohyphomycotic abscess-associated hospitalizations, United States, 2016–2021
Source: PLoS Negl Trop Dis. 2025 Sep 4;19(9):e0013499. doi: 10.1371/journal.pntd.0013499 (PMC12419653; doi:10.1371/journal.pntd.0013499)
Supplement: S1 Table — (DOCX) [file pntd.0013499.s001.docx]

**S1 Table**: ICD-10 codes for chromoblastomycosis and phaeohyphomycotic abscess and other conditions

| **Condition** | **ICD-10-CM code(s)** |
| --- | --- |
| Alcohol-related disorders | F10, G62.1, G31.2, G72.1, I42.6, K29.2, K70.0–K70.4, K70.9, K85.2, K86.0 |
| Autoimmune inflammatory disease | K50–K51, L40, M02.3, M05–M06, M08, M33, M35.2, M45 |
| Chronic kidney disease | N18 |
| Chromoblastomycosis and phaeohyphomycotic abscess | B43 |
| Cutaneous chromoblastomycosis | B43.0 |
| Phaeohyphomycotic brain abscess | B43.1 |
| Subcutaneous phaeohyphomycotic abscess and cyst | B43.2 |
| Other forms of Chromoblastomycosis | B43.8 |
| Chromoblastomycosis, unspecified | B43.9 |
| COPD | J41–J44 |
| Diabetes | E08–E13 |
| Dyslipidemia | E78 |
| Hematologic malignancy | C81–C95 |
| HIV infection | B20, Z21 |
| Hypertension | I10 |
| Liver disease | K70–K77 |
| Lymphedema | I89.0 |
| Solid malignancy | C00–C81 (excluding C44) |
| Solid organ or stem cell transplant | Z94 (excluding Z94.5–Z94.7) |
