# Supplementary material for: Chromoblastomycosis and phaeohyphomycotic abscess-associated hospitalizations, United States, 2016–2021
Source: PLoS Negl Trop Dis. 2025 Sep 4;19(9):e0013499. doi: 10.1371/journal.pntd.0013499 (PMC12419653; doi:10.1371/journal.pntd.0013499)
Supplement: Attachment — Submitted filename: CBM PHM comments.docx [file pntd.0013499.s002.docx]

Chromoblastomycosis (CBM) and phaeohyphomycosis (PHM)

represent two poles of a wide spectrum of diseases caused by diverse species of

dematiaceous fungi. Clinically, the boundaries of the spectrum are imprecise. In both diseases, patients may present abscess, plaque, nodular and ulcerative lesions. Some etiological agents of CBM and PHM may cause both types of infections. Both infections can be found in either immunocompetent or immunosuppressed hosts, but CBM is prevalent in immunocompetent patients, while PHM is mostly not.

In this manuscript, data from the Healthcare Cost and Utilization Project’s National Inpatient Sample, were analyzed regarding age group, sex, census region, season of hospital admission and associated risk factors. Regarding the clinical forms, due to the retrospective data bank analysis, Regarding the clinical forms, they are poorly described and confused, due to the retrospective data collection.

General comments

The title (Chromoblastomycosis and Phaeohyphomycotic Abscess-Associated Hospitalizations, United States, 2016–2021), is not clear. Both diseases CBM and PHM may present several types of lesions beyond “abscesses”.

The number of CBM cases (122), seems to be too high, based on the burden of this disease in the USA: Santos DWCL, et al PLoS Negl Trop Dis 15(8): e0009611. <https://doi.org/10.1371/journal.pntd.0009611> and American published case report(s).

Please comment.

The main issue of this retrospective analysis may impact some conclusions:

‘Substantial in-hospital mortality and complications like lymphedema can occur from chromoblastomycosis and phaeohyphomycotic abscesses”
Differently from implantation PHM, CBM is a chronic and indolent implantation mycoses, and it is important for its mobility but not for mortality.

Specific comments.

In some tables the term “chromomycosis” is used. Please use “chromoblastomycosis” or explain that chromomycosis was used as a “key word key word Search”
