# Supplementary material for: Chromoblastomycosis and phaeohyphomycotic abscess-associated hospitalizations, United States, 2016–2021
Source: PLoS Negl Trop Dis. 2025 Sep 4;19(9):e0013499. doi: 10.1371/journal.pntd.0013499 (PMC12419653; doi:10.1371/journal.pntd.0013499)
Supplement: Attachment — Submitted filename: renamed_c2805.docx [file pntd.0013499.s003.docx]

The paper entitled *'Chromoblastomycosis and Phaeohyphomycotic Abscess–Associated Hospitalizations, United States, 2016–2021'* is of great relevance for the epidemiologic reporting of a disease that has garnered increasing attention in the medical and mycological literature. It is a concise, objective, and accessible manuscript. However, a few methodological considerations warrant the authors' attention to ensure that the data presented are accurate, reliable, and truly reflective of the disease burden.

This is a review of a nationally significant database, highlighting a neglected mycosis being addressed within a developed country—often associated with impoverished regions and populations facing socio-economic disadvantages.

- It is important to emphasize in the Background section that this is not merely a report of subcutaneous disease. In immunocompromised individuals, PHM may present with a more invasive clinical course, affecting deep organs such as the lungs and central nervous system, and may also lead to fungemia.

- I suggest that the authors standardize the terminology by consistently using either 'black fungi' or 'melanized fungi.' It would be preferable to avoid the term dematiaceous fungi.

- It is critically important to clarify that the methodology applies exclusively to hospitalized patients—an inclusion criterion that predominantly reflects cases of phaeohyphomycosis, especially among individuals with comorbidities, as described by the authors. Chromoblastomycosis rarely requires hospitalization for diagnosis, and this selection bias significantly impacts the results and should be explicitly acknowledged to the readers.

- Furthermore, the search strategy relies on some diseases codes in the U.S. system (ICD-10-CM codes), which does not allow for a clear distinction between chromoblastomycosis (CBM) and phaeohyphomycosis (PHM). The ICD-10-CM code B43.8 refers to 'other forms' of chromoblastomycosis (CBM), which have historically included cases mistakenly classified as phaeohyphomycosis (PHM) or as visceral CBM (e.g., involving the CNS, brain, lungs, etc.). It is important to clarify this point to avoid the misinterpretation that there is an unexpectedly high number of CBM cases in the United States—a region where PHM is, in fact, more commonly reported.

- In the table, the subtypes of ICD-10-CM code B43 are listed, including 'Chromomycosis, unspecified' and 'Other forms of chromomycosis,' which together account for 100 clinical cases. It is important to note that these entries may, in fact, correspond to cases of phaeohyphomycosis (PHM) rather than true chromoblastomycosis (CBM), due to historical misclassification and overlap in coding. This distinction is essential to avoid overestimating the burden of CBM in regions like the United States, where PHM is far more prevalent.

- Wangiella is an outdated genus name that has been reclassified, with *Wangiella dermatitidis* now recognized as *Exophiala dermatitidis*, according to current fungal taxonomy.

- Table 2 is excellent and provides highly valuable data in this context. However, as the authors may note, the number of reported CBM cases in patients with chronic kidney disease (n = 65) and solid malignancies (n = 30) appears disproportionately high. These comorbidities are more commonly associated with phaeohyphomycosis (PHM) rather than chromoblastomycosis (CBM). It might be worth investigating whether this subgroup of patients was primarily diagnosed under ICD-10-CM codes B43.8 ('Other forms of chromomycosis') or B43.9 ('Chromomycosis, unspecified'), which may reflect misclassified PHM cases.

This is an important point for reflection, especially considering the ongoing efforts by the CBM study group to ensure that published data over the past decade reflect an accurate, consistent, and realistic epidemiological picture of this disease.

I believe that the majority of cases in this dataset likely represent PHM. Given these limitations, it may be appropriate to revise the manuscript title to: “Melanized Fungal Infections in the United States: An Analysis of Hospitalizations”.

I believe this manuscript should be **considered for publication pending minor revisions**, as outlined above. Addressing these methodological and terminological issues will enhance the clarity, accuracy, and scientific contribution of the work.
