# Supplementary material for: Chromoblastomycosis and phaeohyphomycotic abscess-associated hospitalizations, United States, 2016–2021
Source: PLoS Negl Trop Dis. 2025 Sep 4;19(9):e0013499. doi: 10.1371/journal.pntd.0013499 (PMC12419653; doi:10.1371/journal.pntd.0013499)
Supplement: Attachment — Submitted filename: PloS NTD_Phaeo & Chromo HCUP_Response to Comments.docx [file pntd.0013499.s005.docx]

June 27, 2025

Dear Editors and Reviewers,

Thank you for your kind remarks and thoughtful reviews of our manuscript entitled “Chromoblastomycosis and Phaeohyphomycotic Abscess-Associated Hospitalizations, United States, 2016–2021” (PNTD-D-25-00604). We appreciate the opportunity to revise and resubmit the manuscript to *PLOS Neglected Tropical Diseases*.

We have revised the manuscript and addressed the reviewer comments below, point-by-point. We highlighted all revisions in the enclosed draft. Please do not hesitate to let us know if our responses need clarification or if the revised manuscript requires further editing.

All authors have seen and approved the manuscript and contributed substantially to the work. This manuscript has not been previously published nor is it being considered for publication elsewhere.

Thank you again for considering our revised manuscript.

Sincerely,

Dallas Smith, PharmD, MAS

Mycotic Diseases Branch, Centers for Disease Control and Prevention

rhq8@cdc.gov

EDITOR AND REVIEWER COMMENTS

Reviewer #1:

Thank you for this opportunity to review this report. Smith and colleagues analyze U.S. hospitalizations related to chromoblastomycosis and phaeohyphomycotic abscesses—rare fungal infections caused by dematiaceous fungi—using national inpatient data from 2016 to 2021. An estimated 690 hospitalizations occurred over the six-year period, with rates highest in 2016 and lowest in 2020. Hospitalization rates were higher among males, older adults (≥65 years), and in the Northeast and South regions. Common comorbidities included hypertension, diabetes, dyslipidemia, and chronic kidney disease. Severe outcomes included lymphedema (5%) and in-hospital death (3%), with an average hospital stay of nearly 10 days.

The aim of this study was to enhance understanding of the epidemiology and clinical outcomes of these rare fungal infections, and thereby highlight the need for increased awareness. I do believe that the authors have been able to accomplish this aim to a certain extent. This study provides important baseline data that can inform future surveillance and management efforts. Some notable strengths include novelty, relevance, and use of a large national dataset increasing the generalizability of findings, coupled with balanced discussion.

While this manuscript is generally well-written, with clear articulation of objectives, methods, and limitations, there are areas in this paper where clarification, improved organization, and enhanced precision could strengthen the work. Please see comments below for specific concerns.

Author lay Summary

When you search black fungi online, results show mucormycosis, as opposed to chromoblastomycosis and phaeohyphomycosis, which is what this research is about. Consider changing black fungi to another term, such as melanized fungi or pigmented fungi.

- *Thank you for flagging this. We agree that “black fungi” is a misnomer, and have changed to “dematiaceous fungi.” We avoided using “melanized fungi,” as certain fungi, such as Cryptococcus, have melanin, but are much different than fungi that cause chromoblastomycosis and phaeohyphomycosis.*

Introduction

The background effectively introduces the clinical relevance of chromoblastomycosis and phaeohyphomycotic abscesses, noting their rarity and the knowledge gap in U.S. epidemiology. There have been multiple mentions of phaeohyphomycosis affecting the brain but disseminated phaeohypomycosis is also relatively common. It would be helpful to briefly mention the full clinical spectrum of disease by discussing other organ systems besides skin, subcutaneous tissue and brain it involves. This will help readers to contextualize the importance of studying these infections.

- *We have added language in the introduction to reflect that phaeohyphomycotic abscesses are a form of phaeohyphomycosis and added commentary about disseminated disease caused endocarditis and pulmonary infections.*

Methods

• Second paragraph – at the end of the sentence, include the study period *for example, from January 2016 to December 2021. Also, consider explaining here why disseminated phaeohyphomycoses was not a part of this study, instead of outlining coding limitations at the very end.

- *We have moved the limitation mentioning the inability to capture disseminated phaeohyphomycoses, other than brain abscesses, to the methods section as suggested by the reviewer. We have also added the study period at the end of the second paragraph.*

• The link for Census region can be moved to references.

- *We have moved the Census website link and made it into a reference.*

• If data is available, it would be helpful to add (1) the antifungal/s used and (2) % of patients undergoing surgical resection. Presenting mortality, length of stay, or complications stratified by diagnosis group (chromoblastomycosis vs. phaeohyphomycotic abscess) could add more clinical value, even if limited by small sample sizes as mortality is generally substantially lower in chromoblastomycosis.

- *Unfortunately, the dataset that we used (HCUP) does not contain information about medications, but we agree that this would be useful data to have. There are many ICD-10-PCS codes for surgeries, and it would be very difficult to determine which ones were related to the fungal infection.*
- *In table 2, we have already stratified mortality, length of stay, and comorbidities by chromoblastomycosis and phaeohyphomycotic abscesses. Unfortunately, the n was too small to display for stratified mortality based on the data source’s requirements. As the reviewer suggested, length of hospital stay was longer for phaeohyphomycotic abscesses. Interestingly, all 35 patients experiencing lymphedema were in the chromoblastomycosis cohort, none were in the phaeohyphomycotic abscesses cohort.*

• Please address typographical redundancy – “B43.8 and B43.8.”

- *Thank you for catching this error. The second “B43.8” should be “B43.9”. We have updated the text accordingly.*

• It would be helpful to add the reference to the SAS 9.4 survey procedure.

- *We have added the specific procedures used in the text: PROC SURVEYFREQ and PROC SURVEYMEANS*

Results

• “Rates were highest in 2016 (0.5/1,000,000) and lowest in 2020 (0.2/1,000,000). Overall, higher hospitalization rates occurred among males (0.4/1,000,000) versus females (0.3/1,000,000) (Table 2). Rates increased with age, with patients aged ≥65 years having the highest rate (0.9/1,000,000). The Northeast had the highest hospitalization rate (0.5/1,000,000) followed by the South (0.4/1,000,000).” - consider rephrasing these sentences as they are the same as abstract. Also, would add (Table 2) after the first sentence instead of the second sentence.

- *We have moved “Table 2” to the first sentence instead of the second sentence. Also, as we are describing results as succinctly as possible, we would like to keep the text similar to the abstract for ease of reading and reinforcement of the data.*

• “By season admitted, more patients were admitted in Fall (29%) which was higher (37%) for chromoblastomycosis alone. Summer (28%) had the largest proportion of admissions for phaeohyphomycotic abscesses.” – consider rephrasing this sentence as it is unclear and can be easily misinterpreted.

- *We have rephrased this sentence to read, “By season of hospital admission, most hospitalizations for chromoblastomycosis alone occurred in Fall (37%). Summer (28%) had the largest proportion of admissions for phaeohyphomycotic abscesses.”*

Discussion

• Change Discussions to Discussion

- *We have changed accordingly.*

• Lymphedema is listed as comorbidities in the abstract, results and table. However, in the discussion it is listed as a complication. Would urge the authors to be consistent. For the table, consider moving lymphedema out from comorbidity and add another column as complication and list lymphedema there.

- *In the table, we have changed the category, “Comorbidities” to “Concurrent diagnoses” to be more consistent with the terminology. As we are relying on ICD-10 codes, we can only determine that the patient received these diagnosis codes during the same hospital stay.*

• Please explain what you mean by – “Our analysis provides a baseline to monitor hospitalizations and mortality along with changes in comorbidities that may impact these outcomes.” What changes in comorbidities?

- *For clarity, we have changed comorbidities to risk factors*

• “Chromoblastomycosis and phaeohyphomycotic abscesses can have long-term complications, including lymphedema, secondary bacterial infections, squamous cell carcinoma, and tissue fibrosis (14,15).” – Would be helpful to evaluate what % of patients in your study developed secondary bacterial infections, as I understand amputation, SCC, and tissue fibrosis can occur after discharge as outlined in your limitation.

- *We agree that being able to capture the percent of patients that developed secondary bacterial infections would be helpful in describing the true burden of disease, it would be very difficult to tease out bacterial infections related to the fungal infection through ICD-10 codes.*

• “Other concurrent conditions seen in our study -- diabetes, solid malignancy, and solid organ transplants -- were also reported as common risk factors in other studies on chromoblastomycosis and phaeohyphomycotic abscesses (6,8)” – would move this section above complications and separate comorbidities from complications.

- *We have reframed the methods, results, and tables to reflect that these are concurrent diagnoses (including lymphedema), thus, we would like to proceed with discussing in the same paragraph as is to avoid attempting to distinguish between comorbidities and complications with the limited data interpretations.*

• The phrase “chromoblastomycosis and phaeohyphomycotic abscess-associated hospitalizations” is repeated excessively; consider using abbreviations or alternate phrasing for brevity once the terms are defined.

- *We have modified the text so that chromoblastomycosis and phaeohyphomycotic abscess-associated hospitalizations is only mentioned a maximum of one time per paragraph in the discussion. As many readers are unfamiliar with the abbreviations of these diseases, we prefer not use them.*

Tables

Table 1

• What was the difference between other forms of chromomycosis and Chromomycosis, unspecified? Can consider explaining in methods.

- *B43.8 would be for if the subtype is known but doesn't fall into any of the other specific sub-codes. B43.9 is for if the provider/coder doesn't know or doesn't specify which subtype it is.*

• The total of individual percentages is not adding to 100%

- *The total individual percentages add up to 99%, which is due to rounding. The number of cases adds up to 690.*

• At the end of the title of table 1, would say – by B43 subtype “of ICD-10-CM code”, 2016 to 2021; as it’s not clear what B43 subtype entails if you simply read the title of the table

- *We agree and have added this to the title and in the table text. We have also included the individual subcodes next to the name*.

• Please spell out coI%. If it is confidence interval, include 95% confidence intervals for key rates if possible.

- *We have spelled out column percentage; these are not confidence intervals*.

Table 2

• The total of individual percentages is not adding to 100% for several cells

- *The individual percentages for each variable may not add up to 100% due to rounding or suppression of data indicated by the asterisk.*

• Please spell out col%. If it is confidence interval, include 95% confidence intervals for key rates if possible.

- *We have added an abbreviations footnote to the table that spells out col % and other abbreviations used. This is not a confidence interval.*

Inclusion of at least one epidemiological figure or graph (e.g., annual trends, geographic variation) would improve accessibility and visual engagement.

- *We agree that these figures would be great data visualization tools, but the data are such small numbers that we don’t believe they would bring any meaningful addition to the manuscript.*

**Reviewer #2:**

Chromoblastomycosis (CBM) and phaeohyphomycosis (PHM) represent two poles of a wide spectrum of diseases caused by diverse species of dematiaceous fungi. Clinically, the boundaries of the spectrum are imprecise. In both diseases, patients may present abscess, plaque, nodular and ulcerative lesions. Some etiological agents of CBM and PHM may cause both types of infections. Both infections can be found in either immunocompetent or immunosuppressed hosts, but CBM is prevalent in immunocompetent patients, while PHM is mostly not.

In this manuscript, data from the Healthcare Cost and Utilization Project’s National Inpatient Sample, were analyzed regarding age group, sex, census region, season of hospital admission and associated risk factors. Regarding the clinical forms, due to the retrospective data bank analysis, Regarding the clinical forms, they are poorly described and confused, due to the retrospective data collection.

General comments

The title (Chromoblastomycosis and Phaeohyphomycotic Abscess-Associated Hospitalizations, United States, 2016–2021), is not clear. Both diseases CBM and PHM may present several types of lesions beyond “abscesses”.

- *Thank you very much for this comment. Due to the limitations of using billing codes (ICD-10 codes), we were only able to capture chromoblastomycosis and phaeohyphomycotic abscesses, specifically. We were not able to capture other forms of disseminated phaeohyphomycosis as this is included in a non-specific billing code. We acknowledge this in our methodology (it was previously in our limitations). We also included more commentary about phaeohyphomycosis in the introduction even though we weren’t able to examine data for all types of dissemination (only brain abscesses).*

The number of CBM cases (122), seems to be too high, based on the burden of this disease in the USA: Santos DWCL, et al PLoS Negl Trop Dis 15(8): e0009611. https://doi.org/10.1371/journal.pntd.0009611 and American published case report(s).

Please comment.

- *The chromoblastomycosis global burden study was a comprehensive literature review of cases diagnosed globally, including in the United States. This study was extremely well-done, but it relied on publications alone. Our study utilized billing codes (ICD-10 codes) to determine cases. Many cases, not only the United States, of chromoblastomycosis are not published, thus, underestimating the true burden of disease without proper surveillance.*

The main issue of this retrospective analysis may impact some conclusions:

‘Substantial in-hospital mortality and complications like lymphedema can occur from chromoblastomycosis and phaeohyphomycotic abscesses”

Differently from implantation PHM, CBM is a chronic and indolent implantation mycoses, and it is important for its mobility but not for mortality.

- *We have added specific comments in the discussion text that teases out that phaeohyphomycotic abscesses can lead to mortality while chromoblastomycosis often causes morbidity and long-term complications. Interestingly in our study, 14% of hospitalizations with chromoblastomycosis experienced lymphedema while none with phaeohyphomycotic abscesses did.*

Specific comments.

In some tables the term “chromomycosis” is used. Please use “chromoblastomycosis” or explain that chromomycosis was used as a “key word key word Search”

- *We agree these comments. The actual ICD-10 code uses the terminology, “chromomycosis”, but we have changed it to chromoblastomycosis to be consistent with the text and the correct terminology.*

**Reviewer #3:**

The paper entitled 'Chromoblastomycosis and Phaeohyphomycotic Abscess–Associated Hospitalizations, United States, 2016–2021' is of great relevance for the epidemiologic reporting of a disease that has garnered increasing attention in the medical and mycological literature. It is a concise, objective, and accessible manuscript. However, a few methodological considerations warrant the authors' attention to ensure that the data presented are accurate, reliable, and truly reflective of the disease burden.

This is a review of a nationally significant database, highlighting a neglected mycosis being addressed within a developed country—often associated with impoverished regions and populations facing socio-economic disadvantages.

- It is important to emphasize in the Background section that this is not merely a report of subcutaneous disease. In immunocompromised individuals, PHM may present with a more invasive clinical course, affecting deep organs such as the lungs and central nervous system, and may also lead to fungemia.

*- Thank you for this comment, and we completely agree. We have updated the background section to include a specific sentence on the other forms of phaeohyphomycosis (even though they weren’t able to be assessed in our study)*

- I suggest that the authors standardize the terminology by consistently using either 'black fungi' or 'melanized fungi.' It would be preferable to avoid the term dematiaceous fungi.

*- We prefer to use the term “dematiaceous fungi” for a number of reasons. The term “black fungi,” especially for clinicians, often invokes thoughts of mucormycosis. In fact, if black fungi is googled, mucormycosis is first to appear. The term “melanized fungi” is a bit too broad for fungi that cause chromoblastomycosis and phaeohyphomycosis. For example, Cryptococcus neoformans has melanin but does not lead to these diseases. Most medical mycology textbooks continue to refer to chromoblastomycosis/phaeohyphomycosis causing fungi as dematiaceous fungi as well.*

- It is critically important to clarify that the methodology applies exclusively to hospitalized patients—an inclusion criterion that predominantly reflects cases of phaeohyphomycosis, especially among individuals with comorbidities, as described by the authors. Chromoblastomycosis rarely requires hospitalization for diagnosis, and this selection bias significantly impacts the results and should be explicitly acknowledged to the readers.

*- In the methodology section, we have already stated that this dataset only includes hospitalizations. However, in the limitations sections, we reinforce this and state that this likely undercounts the true burden of chromoblastomycosis in the United States.*

- Furthermore, the search strategy relies on some diseases codes in the U.S. system (ICD-10-CM codes), which does not allow for a clear distinction between chromoblastomycosis (CBM) and phaeohyphomycosis (PHM). The ICD-10-CM code B43.8 refers to 'other forms' of chromoblastomycosis (CBM), which have historically included cases mistakenly classified as phaeohyphomycosis (PHM) or as visceral CBM (e.g., involving the CNS, brain, lungs, etc.). It is important to clarify this point to avoid the misinterpretation that there is an unexpectedly high number of CBM cases in the United States—a region where PHM is, in fact, more commonly reported.

*- We acknowledge these coding limitations in our discussion and realize that some phaeohyphomycotic abscesses were miscoded as chromoblastomycosis. Our results do reflect that phaeohyphomycotic abscesses (and our analysis does not include disseminated cases outside of the brain) are more than twice as common as chromoblastomycosis in the United States, reflecting the reviewer’s statement above. However, chromoblastomycosis still occurs in the United States and may be more common than historically thought to be (although, still rare).*

- In the table, the subtypes of ICD-10-CM code B43 are listed, including 'Chromomycosis, unspecified' and 'Other forms of chromomycosis,' which together account for 100 clinical cases. It is important to note that these entries may, in fact, correspond to cases of phaeohyphomycosis (PHM) rather than true chromoblastomycosis (CBM), due to historical misclassification and overlap in coding. This distinction is essential to avoid overestimating the burden of CBM in regions like the United States, where PHM is far more prevalent.

*- Please see comment above. Our data, does in fact, show that phaeohyphomycotic abscesses alone (not including disseminated disease) are twice as common as chromoblastomycosis in the United States. Miscoding may have occurred as the reviewer suggested, and we acknowledged this in the limitations.*

- Wangiella is an outdated genus name that has been reclassified, with Wangiella dermatitidis now recognized as Exophiala dermatitidis, according to current fungal taxonomy.

*- Thank you for catching this! We have updated the introduction accordingly. We have also removed Bipolaris as the clinically-relevant species have been reclassified as Curvularia species.*

- Table 2 is excellent and provides highly valuable data in this context. However, as the authors may note, the number of reported CBM cases in patients with chronic kidney disease (n = 65) and solid malignancies (n = 30) appears disproportionately high. These comorbidities are more commonly associated with phaeohyphomycosis (PHM) rather than chromoblastomycosis (CBM). It might be worth investigating whether this subgroup of patients was primarily diagnosed under ICD-10-CM codes B43.8 ('Other forms of chromomycosis') or B43.9 ('Chromomycosis, unspecified'), which may reflect misclassified PHM cases.

*- Chromoblastomycosis has been reported in the literature in patients with chronic kidney disease and solid organ malignancies. While our data could reflect miscoding of these diseases (which we have acknowledged in the limitations section), it could also reflect different epidemiology seen in the United States vs in other parts of the world. For example, in the United States, more than 1/7 adults are estimated to have chronic kidney disease. The United States also has the highest rates of organ transplants in the world. Since the literature has shown that chromoblastomycosis can occur in these patients, the data we describe may be from a larger population in the US with these comorbidities compared to the many parts of the world where chromoblastomycosis occurs commonly but also where rates of transplants and CKD are much lower at a population level.*

This is an important point for reflection, especially considering the ongoing efforts by the CBM study group to ensure that published data over the past decade reflect an accurate, consistent, and realistic epidemiological picture of this disease.

I believe that the majority of cases in this dataset likely represent PHM. Given these limitations, it may be appropriate to revise the manuscript title to: “Melanized Fungal Infections in the United States: An Analysis of Hospitalizations”.

- *Our data does demonstrate that the majority of the cases are phaeohyphomycotic abscesses as the reviewer mentions. We don’t think using the terminology melanized fungi is appropriate as some fungi that don’t cause these diseases have melanin (e.g., Cryptococcus neoformans).*

I believe this manuscript should be considered for publication pending minor revisions, as outlined above. Addressing these methodological and terminological issues will enhance the clarity, accuracy, and scientific contribution of the work.
